# Artificial Pneumothorax in the Treatment of Pulmonary Tuberculosis

**Published:** 1911-01

**Authors:** Mary E. Lapham

**Affiliations:** The Highlands Camp Sanatorium, Highlands, N. C.


### Journal-Record of Medicine

Successor to Atlanta Medical and Surgical Journal, Established 1855, and Southern Medical Record, Established 1870.

OWNED BY THE ATLANTA MEDICAL JOURNAL CO.

**Published Monthly**

*Official Organ Fulton County Medical Society, State Examining Board, Presbyterian Hospital, Atlanta, Birmingham and Atlantic Railroad Surgeons' Association, Chattahoochee Valley Medical and Surgical Association, Etc.*

EDGAR G. BALLENGER., M. D., Editor.
BERNARD WOLFF, M. D., Supervising Editor.
A. W. STIRLING, M. D., C. M., D. P. H., J. S. HURT, B. Ph., M. D.
GEO. M. NILES, M. D., W. J. LOVE, M. D., (Ala.); Associate Editors.
E. W. ALLEN, Business Manager.

COLLABORATORS

Dr. W. F. WESTMORLAND, General Surgery.
F. W. McRAE, M. D., Abdominal Surgery.
H. F. HARRIS, M. D., Pathology and Bacteriology.
E. B. BLOCK, M. D., Diseases of the Nervous System.
MICHAEL HOKE, M. D., Orthopedic Surgery.
CYRUS W. STRICKLER, M. D., Legal Medicine and Medical Legislation.
E. C. DAVIS, A. B., M. D., Obstetrics.
E. G. JONES, A. B., M. D., Gynecology.
R. T. DORSEY, Jr., B. S. M. D., Medicine.
L. M. GAINES, A. B., M. D., Internal Medicine.
J. N. LeCONTE, M. D., Disease of the Stomach and Intestines.
L. B. CLARKE, M. D., Pediatrics.
EDGAR PAULIN, M. D., Opsonic Medicine.
THEODORE TOEPEL, M. D., Mechano Therapy.
R. R. DALY, M. D., Medical Society.
A. W. STIRLING, M. D., etc., Diseases of the Eye, Ear, Nose and Throat.
BERNARD WOLFF, M. D., Diseases of the Skin.
E. G. BALLENGER, M. D., Diseases of the Genito-Urinary Organs.

VOL. LVI. JANUARY, 1911 NO. 10.

### ARTIFICIAL PNEUMOTHORAX IN THE TREATMENT OF PULMONARY TUBERCULOSIS.

MARY E. LAPHAM, M. D., OF THE HIGHLANDS CAMP SANATORIUM, HIGHLANDS, N. C.

An artificial pneumothorax is the latest and most scientific method of treating cases of pulmonary tuberculosis, that do not recover under symptomatic and tuberculin treatment. It is practically the application of an elastic compress to the entire surface of the diseased lung, by filling the pleural cavity with nitrogen until sufficient pressure is obtained to render the expansion of the lung impossible—as evidenced by the complete absence of breath sounds.

The introduction of the nitrogen into the pleural cavity is as simple and easy as the aspiration of a pleural effusion and is performed in much the same way. The needle is introduced into the pleural cavity, the nitrogen turned on, the needle withdrawn and the puncture sealed with collodion. What does the presence of nitrogen in the pleural cavity accomplish? Pressure—an elastic, persistent, steady. even pressure over all the surface of the lung, that gradually crowds it up together into the smallest possible space and holds it firmly there, until all the liquifying, caseating, decomposing contents are removed, the lung clean and dry, and the tubercular lesions converted into durable scar-tissue, by an excessive overgrowth of connective tissue, while the sound portions of the lung remain unaffected and able to expand and resume their function after the pressure is removed. The complete compression of the lung obliterates all tubercular processes, crushes them out, and, by a subsequent organization of their lesions, obtains a permanent anatomical recovery that is the surest safeguard against relapse.

A tubercular lung may pass the stage when it is amenable to medical treatment and only surgical measures will avail. When we have ulcerating surfaces, collections of pus, leakage from blood vessels, and masses of decomposing materials, is it likely that symptomatic and tuberculin treatment will avail? Would it not seem more rational—more analogous to our treatment of similar conditions in other parts of the body—if we endeavor to remove these abscesses? To clean out, and drain, and help the organization of the diseased tissues into good, clean scar tissue?

If a bloodless operation will accomplish all, and more than the knife can do, is it not worthy of our careful consideration? A simple and inexpensive apparatus is described by Murphy in his "Oration on Surgery," in the J. A. M. A., for 1898. It can readily be made at home by buying a three-fold stop-cock, a glass irrigator and rubber tubing. A graduated nitrogen jar can be made by pasting a strip of adhesive on the bottle and marking the 100 C. C. divisions on it with India ink. Or the apparatus can be bought from Truax, Green & Co., Chicago.

Each time, before using the apparatus, test it to see if the nitrogen flows properly by putting the needle in hot water, raising the reservoir and turning on the nitrogen to see if it bubbles through the water.

The technic of the first filling is concerned with the production of a pleural cavity which normally does not exist. If the pleural surfaces are free there will be no difficulty, for, as soon as negative pressure is reached, air will rush in through the head of the needle and be plainly heard. If, however, the pleural surfaces are adherent, the needle will pass through both simultaneously and enter the lung, while no air will be heard passing through the head of the needle.

Pleural effusions offer the greatest obstacle to the success of the method. If they are universal no pneumothorax can be produced, and if the lung is held out by partial adhesions, only an incomplete pneumothorax can be obtained, since the lung cannot be sufficiently compressed to extinguish the breath sounds. Pleural adhesions are most likely to be absent where good resonance is found over the most normal breath sounds. Avoid the cardiac area and the diaphragm and, if possible, choose a wide intercostal space, not covered by the heavy muscles. Paint the perfectly dry skin with tincture of iodine. Then with the well sterilized fingers of the left hand stretch the skin tightly over the space to be incised, placing the first and second fingers upon the ribs of the interspace. Freeze with ethyl chloride and make a small incision through the skin and down into the muscles—just large enough to admit a medium size aspirating needle, which is thrust in until the pleura is felt to yield and give. If the pleurae are free, air will rush in and be plainly heard. If no sound is heard, withdraw the needle a little and have the patient cough. If necessary, repeat: if still no sound is heard the needle is in the lung and another attempt must be made elsewhere. Possibly not more than an inch or so away the pleurae may be free. In a case of mine, treated by Murphy, after two failures I saw the third attempt succeed within two inches of the first failure, and Forlanini succeeded in one case after making 28 attempts. Now connect the needle with the nitrogen, raise the reservoir and allow about 250 to 400 cc. of nitrogen to enter. Just enough to keep the pleurae apart and make the next filling easy but not enough to cause any discomfort. Throughout the course of the treatment the patient should be as unconscious as possible of the process. Withdraw the needle quickly, seal the incision with collodion and draw the edges together with adhesive. The subsequent fillings are made every other day or twice a week until the lung has become well compressed, with absence of breath sounds, for two months. Then the character of the sounds will indicate how often the filling must be repeated. As the lung is being compressed metallic breathing appears, followed later by a metallic click and still later by a sound as of water dropping. If the lung is not held sufficiently compressed the reverse will be true. The quantity depends upon the size of the chest and the condition of the lung. The more the lung is infiltrated the less it will yield and, therefore, the less nitrogen can be used at one time. The amount of pressure that can be exerted upon the lung is limited by the amount of resistance that the tissues conveying that pressure can offer. The mediastinal pleura is unsupported. Too high an intrapleural pressure will cause leakage into the mediastinum, with very painful, deep-seated emphysemas running up into the neck, down to the diaphragm, and over on the other side.

These deep-seated emphysemas cause an irregular heart action and a sense of suffocation. They are entirely unnecessary, as nothing is gained by forcing the process. An infiltrated lung must be given time to have the extraneous matter removed.

The after-fillings are give through a fine hypodermoclysis needle. After slightly freezing the skin with ethyl chloride this is easily thrust through. Stretch the skin between the fingers, freeze and thrust the needle in slowly, avoiding the ribs, where the contact is painful. If the ribs are very close together you may have to feel for the space after puncturing the skin. When the needle is in the pleural cavity, wait a moment to see if a pleural reflex will occur. The irritation of the pleura, by this passage of the needle, may occur at any filling. The patient says: "I feel badly. My eyes are queer. I cannot see." Then follows an appalling condition. The pupils contract, the respiration shallows, the skin mottles, collapse and failure of the vital centers seem imminent. This state may last thirty-six hours or be over in a few minutes. It is best met by morphine and stimulants—especially breathing ammonia. These attacks do not indicate discontinuance of the treatment. They are forestalled by a preliminary injection of morphine. If the patient is all right, inject from 200 to 400 cc. of nitrogen, but do not depend upon the patient's sensations as to the quantity to be used. A patient is not always immediately conscious of the effects, and enough nitrogen has been given to cause its escape through the tiny puncture wound out into the muscles and subcutaneous tissues with crackling sounds and much discomfort while the patient has asserted that no excessive pressure was felt.

Throughout the treatment avoid the effects of too much pressure. The heart must not be displaced. Give the lung time to yield. The digestion must not be upset. No violent changes should be made, but slowly, gradually, persistently crush out the tubercular processes without any discomfort to the patient, and hold the lung firmly together until the lesions are well organized and further relapses impossible. It will take about a year to insure an anatomical recovery, but clinical recovery is quicker. Nothing in surgery can surpass the gratifying change wrought in a desperate condition by this method. To see the fever and night sweats disappear, appetite and strength come back, the amount of expectoration dwindle to almost nothing, with the complete disappearance of tubercle bacilli, is as gratifying as any surgical recovery.

The theory of immobilizing the lung by nitrogen injected into the pleural cavity was suggested by Forlanini in 1882, and independently again, in 1898, by Murphy, who published a report of five cases treated by this method. Having established its validity and, since it belonged to medicine rather than surgery, Murphy did not continue using it, and so the impression was created that he disapproved of it, which was sufficient to condemn its use in this country. In Europe it has been chiefly tested by its author, Forlanini, of the University of Pavia; Brauer, formerly of Marburg, now head of the great Eppendorfer Institute, and the specialists of Davos: Lucius Spengler, Ludwig, von Muralt, Frey, Phillippi and others.

Reports of hopeless cases that have been cured are already abundant, and a glance at the Literature List in the 4th number of the 14th volume of Brauer's "Beitrage zur Klinik der Tuberkulose," will give an idea of the importance of the subject. Murphy's original article and the report of his assistant, Lemke, are all that have been written in English. The most important foreign articles are by Forlanini, Brauer, Lucius Spengler, Ludwig von Muralt and Saugman.

Brauer has placed the method upon a scientific basis and proven by laboratory studies two important facts. First: compression of the lung favors organization of tubercular lesions just as a thrombus is organized. Starting from the walls of the blood vessels, bronchi, and lymphatics, connective tissue buds shoot out into the injured tissues and bind them firmly together into a cicatricial mass in which no infection can ever flourish—thus securing an anatomical recovery of the most durable nature. Second: Uninjured alveoli show no disposition to adhere even after the lung has been compressed for a year, but readily expand and resume their function after the pressure is removed. Forlanini reports a striking case of a young woman with a large cavity in the right upper lobe. The lung was compressed until the physical signs and X-ray suggested complete organization and the lung was allowed to expand. Fourteen years afterward the woman died of another disease and at the autopsy nothing remained of the former huge cavity but scars, while the rest of the lung was entirely normal. Compression of the lung is indicated in any case of pulmonary tuberculosis that does not do well after a fair trial of symptomatic and tuberculin treatment. This includes several classes, but most especially the desperate, hopeless ones that are doomed. The emaciated, prostrated patients with high fever, exhausting night sweats, and purulent expectoration, with one lung totally involved and riddled with foci of destruction. Many of these have been saved. Another class is unable to make, or to hold recovery, although the lesion is slight. A typical case is that of a young lawyer with only a slight central lesion. The physical signs would clear away and then, on the slightest provocation (a little extra exercise or strain), would return in full vigor. As he had a family to support, we compressed the lung. In two months he was safely at work, after having spent a year with symptomatic and tuberculin treatment.

Women are especially exposed to relapses on account of the vicious influences of menstruation. In some cases there is a marked tendency to hyperemia of the tubercular lesions, leading to transudation, rales and extension of the pneumonic process. The throat fills up, the voice becomes stiff and raucous, the temperature rises, appetite and digestion disappear. Curiously enough these mentrual waves are apt to occur after the patient has made good progress towards recovery. After some months of gratifying gain in weight, strength, and the disappearance of physical signs, the vicious pelvic influence returns with tenfold strength and all is lost at a blow. Hemorrhage, aspiration—pneumonia, wet rales, paroxyomal cough and vomiting all testify to pressure and congestion of the lung. Menstruation extends the pneumonic processes. Tuberculosis upsets mentruation and when this vicious circle is established it is hard to break it up.

Another class is that in which the lesion is apparently very slight, but the temperature keeps up above 38 and the patient cannot take any exercise without bringing on a relapse. These are cases that do not sufficiently declare themselves by physical signs. They go on indefinitely, confusing and puzzling both patient and doctor, because the extent of the lesion, as indicated by physical signs, is out of all proportion to the inability of the patient to recover. Compression of the lung puts these patients on their feet at once.

The contra-indications are: Any complication sufficient in itself to inhibit recovery (such as diabetes), and too great an involvement of the second lung. Lesions of the upper third of the second lung are no contra-indication for two reasons: 1st. If the physical signs are altered breath sounds and rales, they may be transmitted from the other lung and disappear with the absence of breath sounds in the other lung. It is at times impossible to say where the altered breath sounds and rales originate, and so it is impossible to determine by auscultation just how much the second lung is affected.

2nd. The removal of the toxins from the other lung exercises a most favorable influence upon the recovery of the second lung. Many of these cases have recovered.

I have under my care at present, a young woman with the left lung involved down to the third interspace. In the right upper lobe was a cavity with amphoric breathing, dripping rales, etc. Thinking that the high fever and exhaustion might be due to secondary infection of the cavity, we compressed the right lung. In two weeks all cavity sounds disappeared, the walls were approximated, the temperature fell to normal. The patient not only felt no inconvenience, but became stronger, had a better appetite and began to rally from an extreme depression of spirits.

Mrs. A., in bed for a year, temperature 38, hemorrhages, left lung entirely involved, apex of right somewhat affected, bad cough, profuse expectoration: in the left lung there was an area in the second interspace, an inch from the sternum, that rose and fell with inspiration and expiration. Over this area were loud bronchial breathing and large, wet rales with tympany. It seemed dangerous to attempt the compression of this cavity for fear of bursting its thin walls. Very gradually, with only the smallest amounts of air, we compressed the lung, obliterating the cavity and finally getting the lung well held up together at the Hilum. After 18 months in bed with failure to recover, the patient is now cooking her own meals and doing light housework after only two months of an artificial pneumothorax.

The advantages of artificial pneumothorax are:

1st. It saves when all else fails.

2d. It adds an element of precision and certainty to recovery that can be obtained in no other way.

3rd. It is the best safeguard against relapse.

4th. It saves time and money and soon puts a man on his feet so that he can support his family.

### BIBLIOGRAPHY.

Brauer, L., Erfahrungen und Uberlegungen zur Lungenkoll apstherapie. I: Die ausgedehnte extrapleurale Thorakoplastik. Brauer's Beitr. z. Klin. der Tuberkulose. Bd, XII, H. 1. S. 49-154.

Brauer, L. Die Behandlung der einseitigen Lungenphthisis mit kunstl. Pneumothorax. Munch. med. Wochens. Nr. 7, 1906.

Brauer, L. Die Behandlung chronischer Lungenkranheithen durch Lungenkollaps. "Therapie der Gegenwart" Juni, 1908.

Brauer, L. Der therapeutische Pneumothorax. Deutsche med Wochenschr. Nr. 17. 1906.

Brauer, L., Uber Pneumothorax. Programm der Feier des Rektoratswechsels. Marburg a-L 1906.

Brauer, L. Uber kuntlichen Pneumothorax. Kongress fur innere Medizin von. 6 bis 9. April in Wein. Therap. Wochenschr. 1 908. Nr. 33. S. 454.

Brauer, L. Die therapeutische Bedeutung des kunstlichen Pneumothorax. Kongress der Deutschen Gesellshaft fur Chirugie zu Berlin, 21-24 April, 1908. Therap. Wochenschr. 1908. Nr. 29.

Forlanini, C. Verusche mit kunstlichem Pneumothorax bei Lungenphthise. Munch. med. Wochenschr. 1894. Nr. 15.

Forlanini. Primi tentativi die pneumothrace artificiale nella tisi polmonare. Gazzetta medica di Torino, 1894. No. 20 e 21; 17 e 24 Maggio.

Forlanini—Primo caso di tisi polmonare monolaterale avanzata, curato felicemente col Pneumotorace artificiale. Gazetta medica ti Torino 1895. Nr. 44.

Forlanini. C. Zur Behandlung der Lungenschwindsucht durch kunstlich erzeugten Pneumothorax. Deutsche med. Wochenschr. 1906. Nr. 35.

Forlanini. Cura della tisi pulmonare colpneumotorace prodotto artificialmente. Due conferenze tenute alla Associazione sanitari Milanese 1907. Gazetta med. italiana 1907-1908.

Forlanini. Die Indikationen und die Tecknik des kunstlichen Pneumothorax bei der Behandlung der Lungenschwindsucht. Therapie Gegenwart. Nov. u Dez. 1908.

Forlanini. Rivista della Pubblicazioni sul pneumotorace terapeutico. Pavia. Successori Marelli.

Lemke. Pulmonary Tuberculosis treated with nitrogen injections. Journ. Am. Assn. 1899.

Muralt, L., v. Ube die Behandlung schwerer, einseitiger

Lungentuberkulose mit kunstlichen Pneumothorax (mit Illustrationen). Munch. med. Wochenschr. 1909. Nr. 50, 51.

Murphy. Surgery of the Lung. J. A. M. A. July and A. 1898.

Orlandi e Antonini. Guarigione clinica ottenuta colpneumotorace artificiale in dur casi di tuberculosi polmonare. Gazzetta med. ital. 1908. Nr. 42.

Saugman, C. Uber die Anwendung des kunstlichen Pneumothorax in der Lungentuberkulose. Zeitschr. f. Tuberkulose u. Heilstattenwesen. 1908. Bd. XII H. I.

Saugman. Uber kunstlichen Pneumothorax (6 nordischer Kongress fur innere Medizen in Skagen vom. 28-30 Juni, 1909). Intern. Zentralbl. f. d. ges. Tuberkuloseforschung. 1909. Nr. 11 S. 527.

Schmidt, A. Zur Behandlung der Lungenphthise mit kunstl. Pneumothorax. Duetche med. Wochenschr. 1906. Nr. 1.

Schmidt, A. Erfahrungen mit dem kunstlichen Pneumothorax bei Tuberkulose, Bronchiektasien und Aspiration krankheiten. Munch. med. Wochenschr. 1908. Nr. 49.

